# Emerging roles for interleukin-11 in the inflammatory response of intracerebral hemorrhage

**DOI:** 10.3389/fneur.2025.1719102

**Published:** 2025-12-18

**Authors:** Letian Xue, Junrou Zhu, Haiyang Wang, Zheng Wang, Wenhua Yu

**Affiliations:** 1 The Fourth College of Clinical Medicine, Zhejiang Chinese Medical University, Hangzhou, China; 2 Department of Neurosurgery, Affliliated Hangzhou First People’s Hospital, School of Medicine, Westlake University, Hangzhou, China

**Keywords:** intracerebral hemorrhage, IL11, inflammatory response, cytokine, neuroprotection

## Abstract

Intracerebral hemorrhage (ICH) is a severe cerebrovascular disorder with high rates of disability and mortality. Secondary inflammatory responses are recognized as pivotal determinants of patient outcomes and neurological recovery. In recent years, interleukin-11 (IL11), an important member of the IL6 cytokine family, has attracted increasing attention for its role in various inflammatory diseases and neurological injuries. Emerging evidence indicates that IL11 expression undergoes significant alterations following ICH and participates in regulating immune cell activation, cytokine secretion, and blood–brain barrier integrity. Beyond its complex interactions with other inflammatory mediators, IL11 may also exert neuroprotective effects, thereby providing a novel theoretical basis for therapeutic interventions targeting post-ICH inflammation. This review systematically summarizes the expression dynamics, molecular mechanisms, and potential therapeutic implications of IL11 in the context of ICH-associated inflammation, with the aim of offering new insights into strategies for modulating inflammatory responses in ICH patients.

## 1 Introduction

Intracerebral hemorrhage (ICH) is a common yet life-threatening acute cerebrovascular event, characterized by persistently high morbidity and disability rates, posing a serious threat to human health. Brain injury following ICH involves both initial mechanical damage and, more critically, a secondary inflammatory response that drives disease progression. This secondary inflammation leads to disruption of the blood–brain barrier (BBB), cerebral edema, neuronal apoptosis, and neurological dysfunction, thereby exacerbating patient outcomes (1–3). In recent years, increasing research has focused on the mechanistic role of inflammation in the pathological progression of ICH and explored strategies to modulate inflammatory responses to ameliorate brain injury and improve neurological recovery (1, 2, 4). Against this background, identifying novel inflammatory mediators and elucidating their molecular mechanisms has become a major focus of both basic and clinical ICH research.

Interleukin-11 (IL11), an important member of the IL6 cytokine family, was initially recognized for its hematopoietic and anti-inflammatory properties. However, emerging evidence has revealed that the role of IL11 in inflammatory diseases and organ injury is far more complex than previously appreciated. IL11 is not only involved in fibrosis and chronic inflammation but also exerts critical regulatory functions in tissue injury and repair across multiple organs, including the heart, kidneys, lungs, and liver (5–10) (Table 1). In the central nervous system (CNS), IL11 expression and function have increasingly attracted attention. Recent studies demonstrate that astrocyte-derived IL11 modulates the crosstalk between astrocytes and microglia, thereby influencing neuronal injury and inflammatory responses (11). For instance, in sepsis-associated encephalopathy models, reduced IL11 secretion facilitates microglial polarization toward the pro-inflammatory M1 phenotype, aggravating neuronal damage, whereas exogenous IL11 supplementation markedly attenuates inflammation and brain injury, suggesting a potential neuroprotective role of IL11 in central inflammatory regulation (11).

**Table 1:** Pleiotropic roles of IL-11 in pathophysiology across organ systems.

System/organ	Disease/pathological process	Primary role of IL-11
Nervous system	Multiple sclerosis (MS)/Experimental autoimmune encephalomyelitis (EAE)	Neuroprotection, promotes remyelination, immunomodulation
Nervous system	Experimental autoimmune encephalomyelitis (EAE)	Immunomodulation and direct neuroprotection
Nervous system	Brain metastasis from NSCLC	Promotes tumor immune evasion
Nervous system	Alzheimer’s disease (AD)	Neuroprotection
Nervous system	Ischemic brain injury	Anti-inflammatory, antioxidant, neuroprotection
Nervous system	Craniosynostosis	Regulates bone formation and remodeling
Renal system	Acute and chronic kidney disease	Pro-fibrotic, pro-inflammatory
Hepatic system	Alcoholic liver disease/liver injury	Pro-fibrotic, pro-inflammatory
Cardiovascular system	Cardiac fibrosis	Core pro-fibrotic mediator
Respiratory system	Idiopathic pulmonary fibrosis (IPF)	Drives fibroblast activation, chronic inflammation
Reproductive system	Preeclampsia	Inhibits trophoblast differentiation, activates inflammasome
Oncology	Breast cancer, GI cancers	Promotes tumor progression
Systemic	Systemic sclerosis, RA, etc.	Pro-fibrotic, pro-inflammatory
Systemic	Aging and multimorbidity	Drives aging pathology

Although the involvement of IL11 in various inflammatory diseases and organ injuries has been established, its expression dynamics and regulatory mechanisms following ICH remain incompletely understood. Current research has primarily focused on IL11 in peripheral organ fibrosis, chronic inflammation, and tissue repair, whereas systematic analyses of its specific functions and signaling pathways in post-ICH neuroinflammation are still lacking (5–7, 11). With ongoing advances in understanding the mechanisms of ICH-induced inflammation, IL11 has emerged as a potential target for modulating secondary brain injury.

Therefore, this review systematically summarizes and critically analyzes the roles, underlying mechanisms, and potential clinical relevance of IL11 in the inflammatory response after ICH. By integrating recent findings from both experimental and clinical studies, we aim to provide a theoretical foundation and new perspectives for elucidating the molecular mechanisms of post-ICH inflammation, identifying novel therapeutic targets, and advancing individualized precision medicine in ICH.

### 1.1 Mechanisms of the inflammatory response after intracerebral hemorrhage

#### 1.1.1 Initiation and progression of secondary inflammation

Following ICH, hematoma formation results in the extravasation of blood components, including erythrocytes and plasma proteins, into the brain parenchyma. These exogenous factors rapidly activate resident immune cells, particularly microglia and astrocytes. As the primary immune effector cells of the CNS, microglia are among the first to be activated after hematoma formation and subsequently release a wide array of inflammatory mediators. Astrocytes also participate in the inflammatory response by secreting chemokines and cytokines, thereby shaping the inflammatory milieu in concert with microglia (12). These inflammatory mediators directly injure neurons and recruit peripheral immune cells (e.g., neutrophils, monocytes) to the lesion, triggering an inflammatory cascade (1, 13). The infiltration and activation of immune cells further amplify cytokine release, forming a positive feedback loop that perpetuates and expands the inflammatory response.

At the same time, the BBB undergoes severe damage during the inflammatory response. Accumulation of blood components and inflammatory mediators increases BBB permeability, facilitating the entry of harmful substances and immune cells into brain tissue, exacerbating edema and neuronal death (3, 14). BBB disruption not only worsens local inflammation but also renders brain tissue more susceptible to external insults, forming a vicious cycle. Moreover, inflammatory responses activate molecular pathways such as the NLRP3 inflammasome and NF-κB signaling, further promoting apoptosis, oxidative stress, and neuronal injury (15, 16). Thus, the initiation and progression of secondary inflammation constitute a key pathological basis for neurological damage and dysfunction after ICH, highlighting the therapeutic value of targeting inflammatory cascades to improve outcomes.

#### 1.1.2 Roles of major inflammatory cytokines

Inflammation is central to secondary brain injury following ICH, with classical pro-inflammatory cytokines such as IL-1β, IL-6, and TNF-α being markedly upregulated and exerting profound effects on neural injury and repair. Multiple studies have confirmed significant elevations of these cytokines in brain tissue, cerebrospinal fluid, and peripheral blood after ICH, closely correlating with neurological deficits and poor prognosis (17, 18). Pro-inflammatory cytokines including IL-1β and TNF-α activate microglia and astrocytes, driving downstream inflammatory cascades that promote oxidative stress, apoptosis, and BBB disruption (19). For example, IL-1β is strongly associated with cerebral edema and neuronal death, with its upregulation after ICH facilitating NLRP3 inflammasome activation and exacerbating neuronal damage (18).

IL-6 contributes not only to inflammatory amplification but also to extracellular matrix remodeling by modulating glial and parenchymal cell function. While this process may facilitate fibrosis and tissue repair, excessive IL-6 activation can result in glial scar formation, impeding neurological recovery (18, 19). TNF-α, a key inflammatory mediator, promotes apoptosis and oxidative stress, aggravating neuronal damage, while also regulating immune cell recruitment and activation, thereby shaping the inflammatory microenvironment (19, 20). Persistent cytokine upregulation after ICH increases BBB permeability, allowing further infiltration of peripheral immune cells into the CNS and perpetuating a vicious cycle of injury (17, 21).

Collectively, the elevation of cytokines such as IL-1β, IL-6, and TNF-α represents not only hallmarks of post-ICH inflammation but also critical drivers of apoptosis, oxidative stress, and extracellular matrix remodeling. Their dynamic alterations provide a theoretical basis for mechanistic studies and targeted interventions in secondary injury after ICH (18, 19). It is noteworthy that IL-11, another member of the IL-6 family, interacts with these classical cytokines and contributes to the inflammatory network post-ICH, a topic that will be elaborated in Section 2.3.3. Furthermore, beyond parenchymal resident cells, the roles of infiltrating immune cells (e.g., monocytes) in cytokine production and ICH pathology are increasingly recognized and will be discussed in subsequent sections regarding IL-11’s cellular sources.

#### 1.1.3 Impact of inflammation on neurological function

The influence of post-ICH inflammation on neurological function is profound, directly linking molecular and cellular events to clinically relevant deficits. For example, elevated levels of pro-inflammatory cytokines like IL-6 and TNF-α in the CSF or periphery have been correlated with worsened cognitive and functional recovery in ICH patients and experimental models (22). These functional impairments are the ultimate consequence of the inflammatory cascades described in previous sections, which lead to neuronal injury, cerebral edema, and BBB breakdown (23–25). Consequently, sustained elevation of pro-inflammatory cytokines after ICH is strongly associated with neurological impairment, whereas inhibition of inflammation alleviates tissue damage and improves functional outcomes (26–28).

In addition, inflammation influences tissue repair by regulating microglial phenotypes. Pro-inflammatory M1 microglia promote neuronal death and dysfunction, whereas anti-inflammatory M2 microglia facilitate clearance of necrotic cells and hematoma, supporting repair and functional recovery (29–31). Thus, appropriately modulating inflammation to favor M2 polarization while suppressing excessive pro-inflammatory responses may mitigate secondary brain injury and enhance recovery. Preclinical studies have shown that anti-inflammatory drugs, immunomodulation, and even physical approaches such as low-intensity pulsed ultrasound can ameliorate neurological outcomes after ICH (32, 33). However, excessive suppression of inflammation may compromise tissue repair, underscoring the need for a balance between inhibiting detrimental inflammation and promoting beneficial recovery. Overall, inflammation acts as both a driver of injury and a regulator of repair after ICH, making its rational modulation critical for functional recovery.

### 1.2 Biological properties and signaling pathways of IL11

#### 1.2.1 Structure and regulation of IL11 expression

IL11 is an important member of the IL6 cytokine family and belongs structurally to the four-helix bundle proteins, characterized by highly conserved sequences. IL11 is secreted primarily by non-immune cells such as fibroblasts and endothelial cells and is expressed across multiple tissues and organs. It exerts broad physiological functions, including immune regulation, inflammation, cell differentiation, and tissue repair. IL11 expression is tightly regulated by inflammatory stimuli and injury-related signals. For instance, in the tumor microenvironment, fibroblast subpopulations such as IL11^+^ inflammatory-associated fibroblasts upregulate IL11 in response to synergistic activation by IL-1β and TNF-α through the canonical NF-κB pathway, highlighting the close relationship between IL11 production and local inflammation (34).

Animal studies further show that changes in local microenvironments can modulate IL11 expression; for example, nasal commensal *Bacillus velezensis* NSV2 induces IL11 mRNA expression in the nasal mucosa, enhancing the mucosal immune barrier (35). In disease states such as nephrolithiasis and diabetes, IL11 expression is significantly elevated compared with healthy controls and linked to specific cell types, emphasizing its central role in regulating inflammation and injury responses (36). Collectively, IL11 expression is not only induced by inflammatory and injury signals but also finely tuned by local microenvironments and intercellular interactions.

#### 1.2.2 IL11 receptors and signaling mechanisms

IL11 exerts its biological functions by binding to its specific receptor IL11Rα, which recruits the common co-receptor gp130 to form a high-affinity signaling complex. This complex activates multiple downstream pathways, most notably the JAK/STAT3 cascade. Upon IL11 binding to IL11Rα, gp130 mediates phosphorylation of Janus kinases (JAKs), which in turn activate STAT3. Phosphorylated STAT3 translocates into the nucleus, where it regulates gene expression (37, 38). The JAK/STAT3 pathway plays a pivotal role in proliferation, differentiation, survival, and inflammation. For example, IL11 promotes extracellular matrix protein synthesis and cytokine secretion, thereby contributing to tissue repair, fibrosis, and chronic inflammation (37, 39).

In addition to JAK/STAT3, IL11 signaling also activates non-canonical pathways, such as ERK signaling. Recent evidence indicates that IL11 stimulation induces sustained ERK activation, which is strongly associated with transdifferentiation, proliferation, and survival, particularly in fibroblast-to-myofibroblast transition and fibrotic diseases (40–42). Sensitivity to IL11 signaling depends on IL11Rα and gp130 expression, which is high in fibroblasts, hepatocytes, and vascular smooth muscle cells but relatively low in immune cells (37, 43).

The role of IL11 in inflammation is also increasingly recognized. IL11 not only promotes survival and anti-apoptotic signaling through JAK/STAT3 but also regulates expression of inflammation-related genes, thereby shaping the inflammatory microenvironment. In pathological conditions such as cancer, colitis, and autoimmune diseases, activation of the IL11–JAK/STAT3 axis promotes inflammation, tissue remodeling, and even immunosuppression within the tumor microenvironment (38, 44). Thus, through its receptor-mediated signaling pathways, IL11 plays a crucial role in proliferation, differentiation, survival, and inflammation, and represents a potential therapeutic target in related diseases.

**Figure 1:**
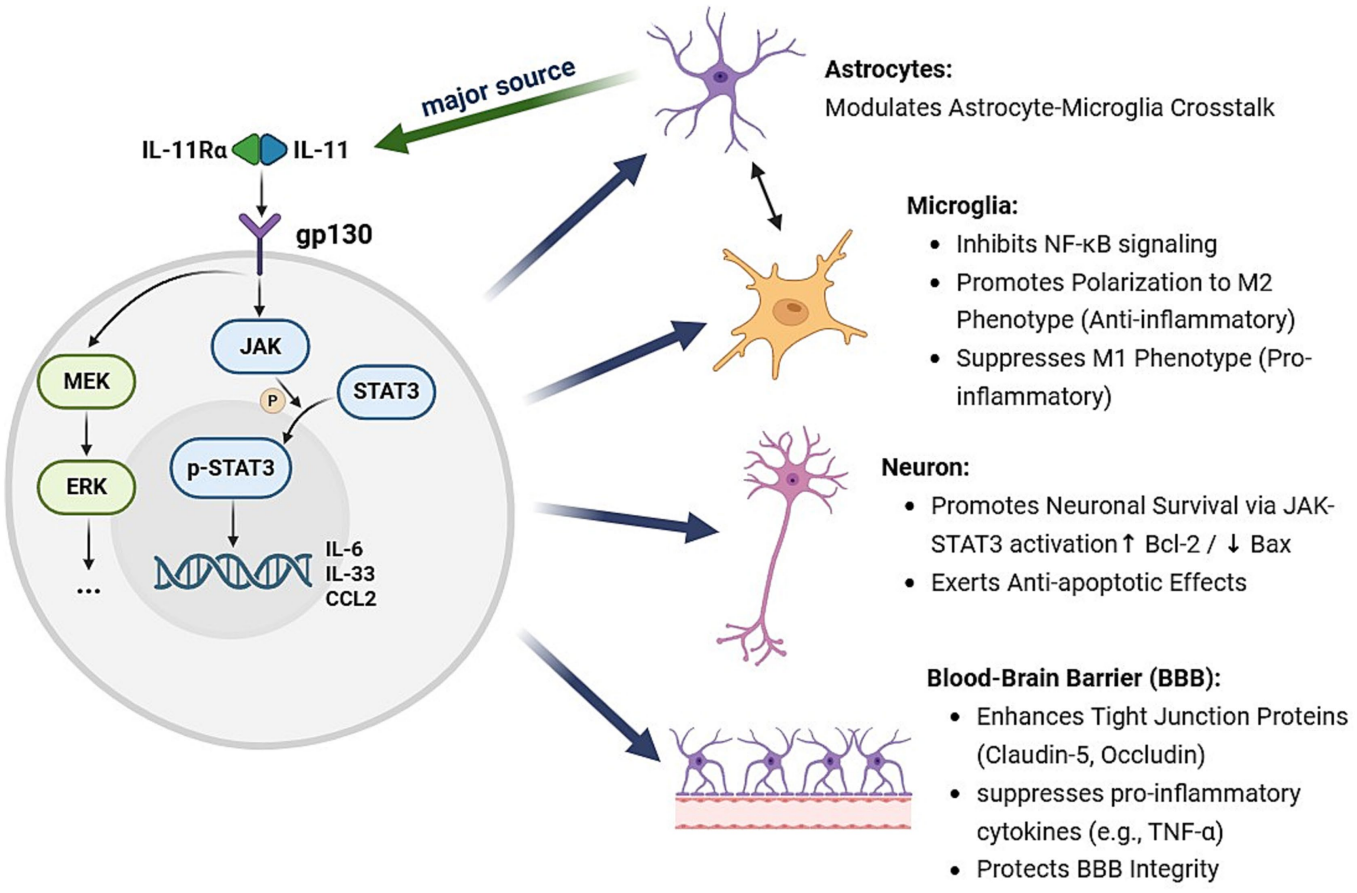
Proposed mechanistic model of IL-11-mediated neuroprotection and immunomodulation following ICH.

As summarized in Figure 1, IL-11 signaling initiates a complex and interconnected intracellular network rather than a linear cascade. Critical to its pleiotropic effects is the extensive cross-talk between its primary downstream pathways. The activation of JAK/STAT3, ERK, and other signaling nodes (e.g., NF-κB) occurs in parallel and exhibits significant synergy. For instance, the TGF-β pathway, a key upstream inducer of IL-11 expression, can simultaneously activate both SMAD and ERK signaling, which in turn reinforces IL-11 production and amplifies the overall signal (45). The integration of these pathways—particularly the dynamic interplay between STAT3 and ERK signaling, their co-modulation by upstream regulators like TGF-β, and the concurrent suppression of NF-κB—collectively underpins the pleiotropic role of IL-11 in ICH pathology (11, 46).

#### 1.2.3 Roles of IL11 in the central nervous system

IL11 expression in the healthy brain is low but is significantly upregulated under conditions of CNS injury or inflammation, suggesting an important regulatory role in pathological states. Studies indicate that IL11 contributes to neuroprotection and glial regulation. For instance, in sepsis-associated encephalopathy (SAE) models, astrocytic IL11 secretion decreases in response to inflammatory stimuli such as LPS, thereby promoting microglial polarization toward the pro-inflammatory M1 phenotype and aggravating neuronal damage. Supplementation with exogenous IL11 attenuates LPS-induced neuronal injury, suggesting that IL11 exerts anti-inflammatory and neuroprotective effects via astrocyte–microglia crosstalk and NF-κB signaling (11).

In brain tumor studies, IL11 expression has been linked to FABP7, a metabolic regulator highly expressed in astrocytes and neural stem cells. FABP7 upregulation promotes IL11 and other immunomodulatory factors, influencing the tumor immune microenvironment (TIME) and correlating with patient prognosis (47). In diffuse white matter injury (dWMI) models, IL11 secreted by mesenchymal stem cells (MSCs) promotes oligodendrocyte maturation and myelination, improving neurodevelopmental outcomes (48). Moreover, chronic alcohol exposure downregulates IL11 mRNA in the rat hippocampus, suggesting a role in neuroinflammation (49). Overall, IL11 contributes to glial regulation, inhibition of inflammation, and promotion of repair in diverse CNS pathologies, underscoring its biological significance and therapeutic potential.

### 1.3 Expression and regulation of IL11 in post-ICH inflammation

#### 1.3.1 Alterations in IL11 expression after ICH

IL11 expression is markedly upregulated after ICH, as demonstrated in both animal models and clinical studies. In common models such as collagenase-induced or autologous blood injection ICH. Data from experimental ICH models (e.g., collagenase-induced or autologous blood injection in rodents) show that IL11 mRNA and protein levels increase significantly, peaking within hours to days after injury. Similarly, clinical analyses reveal elevated IL11 levels in cerebrospinal fluid and serum of ICH patients compared with controls, implicating IL11 as a regulator of post-ICH inflammation.

Immunohistochemical and *in situ* hybridization studies indicate that IL11 is predominantly expressed in glial cells and vascular endothelial cells surrounding the hematoma. Activated glia—particularly astrocytes and microglia—are central players in CNS inflammation. Enhanced IL11 expression in these cells suggests roles in modulating local inflammation and neuroprotection. Vascular endothelial cells, another major IL11 source, upregulate IL11 in response to BBB injury, potentially influencing vascular permeability and immune cell recruitment.

The temporal and spatial dynamics of IL11 expression parallel the course of post-ICH inflammation. Early rapid induction may reflect regulation of acute inflammation, while sustained expression during later stages suggests involvement in repair and resolution. These findings highlight IL11’s dual functions in CNS injury and underscore its potential as a therapeutic target.

While astrocytes and endothelial cells are established sources of IL-11 following ICH, its expression and function within other components of the neurovascular unit warrant consideration. Critically, emerging evidence implicates IL-11 in oligodendrocyte integrity. A study on ellagic acid-induced neuroprotection in acute demyelination identified IL-11 as a key component of the CXCL12/IL-17/IL-11 axis, crucial for limiting mature oligodendrocyte apoptosis (50). This provides direct evidence linking IL-11 signaling to oligodendrocyte survival. In contrast, the role of IL-11 in pericytes, despite their critical importance in BBB stability, remains entirely unexplored in the ICH context and represents a significant gap for future investigation. Elucidating IL-11’s role across this broader cellular network will provide a more comprehensive understanding of its impact on neuroinflammation and repair.

#### 1.3.2 Molecular mechanisms regulating IL11 expression

Data derived primarily from animal models and *in vitro* studies indicate that IL11 expression is modulated by diverse mechanisms within the inflammatory milieu of ICH. For example, studies in rodent models of tissue injury have shown that IL11 expression is modulated by diverse mechanisms within the inflammatory milieu of ICH. Hematoma components, oxidative stress, and inflammatory microenvironments robustly induce IL11. For example, in various tissue injury and fibrosis models, stress signals such as mechanical strain, oxidative stress, and cytokine stimulation upregulate IL11, activating downstream inflammatory and fibrotic processes (51–53). TGFβ represents a canonical pathway for IL11 induction, markedly elevating IL11 mRNA and protein across cell types and activating ERK signaling to promote phenotypic transitions and inflammation (40, 52, 54).

Oxidative stress mediators such as NOX2 contribute to IL11-driven amplification loops, forming a positive regulatory circuit—IL11–ERK–TNC–TLR4–NOX2—that sustains inflammation and fibrosis (55). At transcriptional and post-transcriptional levels, microRNAs and transcription factors also regulate IL11. In alcohol-induced neuroinflammation, downregulation of miR-let7b, miR-96, and miR-155 correlates with decreased IL11 mRNA (49). Transcription factors such as STAT3 and NF-κB enhance IL11 transcription under inflammatory conditions, while cooperation with ERK signaling regulates processes including cell cycle progression, differentiation, and immune evasion in pathologies such as cancer and tissue injury (56–58).

Thus, IL11 regulation involves multilayered mechanisms: injury- and inflammation-related stimuli induce expression via TGFβ–ERK and oxidative pathways, while microRNAs and transcription factors fine-tune transcriptional and post-transcriptional control. Together, these networks shape IL11 dynamics and function in ICH-associated inflammation.

#### 1.3.3 Interactions between IL11 and other inflammatory cytokines

As a key member of the IL6 cytokine family, IL11 occupies a complex position in inflammatory regulation. Evidence indicates that IL11 not only functions independently but also acts synergistically with cytokines such as IL6 and TNF-α to influence the magnitude and persistence of inflammation. In multiple disease models, IL11 and IL6 are co-upregulated and exert synergistic effects in fibrosis, immune activation, and inflammatory cascades. For instance, in intestinal injury models, IL11 and IL6 expression is regulated by TLR4 signaling, and their downregulation exacerbates inflammation (59).

Despite structural similarities, IL6 and IL11 differ in receptor distribution and biological effects. IL6R is primarily expressed in immune cells, whereas IL11RA is highly expressed in hepatocytes and stellate cells. Consequently, their roles in liver disease diverge—IL6 supports protection and regeneration, whereas IL11 promotes inflammation and fibrosis (43).

In CNS inflammation models, reduced IL11 secretion promotes microglial M1 polarization and NF-κB activation, exacerbating inflammation, whereas exogenous IL11 supplementation reverses these effects and mitigates neuronal injury, underscoring its central role in astrocyte–microglia interactions (11). Beyond the CNS, IL11 forms positive feedback loops with cytokines such as IL-1β and IL-17, amplifying inflammatory cascades (60). Moreover, IL11 and cytokines such as TNF-α and IL6 are often co-upregulated in chronic inflammation and fibrosis across organs including the liver, heart, lungs, and kidneys (5, 6, 61).

Although IL11’s interactions with other cytokines are well documented, its precise role within inflammatory networks remains incompletely defined. Some studies suggest that IL11 modulates downstream cytokine expression by activating ERK, STAT3, and NF-κB pathways, thereby regulating immune infiltration, cytokine release, and tissue repair (8, 39, 62). Importantly, IL11’s effects exhibit tissue and cell specificity, with interaction patterns varying across organ systems. Elucidating these dynamic mechanisms will provide a molecular foundation for targeting IL11 in inflammation-related diseases.

### 1.4 IL11-mediated inflammatory responses and neuronal injury

#### 1.4.1 Effects of IL11 on inflammatory cell activation

IL11 plays a crucial role in modulating the activation and phenotypic transformation of inflammatory cells, particularly within the inflammatory microenvironment of CNS disorders such as ICH. Studies have shown that IL11 not only influences the activation states of microglia and macrophages but also exerts bidirectional regulation between pro-inflammatory and anti-inflammatory phenotypes. In microglia, IL11 modulates polarization toward either the M1 (pro-inflammatory) or M2 (anti-inflammatory) phenotype. For example, in sepsis-associated encephalopathy (SAE) models, decreased astrocytic IL11 secretion promotes microglial polarization toward the M1 phenotype, activates NF-κB signaling, and aggravates neuronal damage. Conversely, exogenous IL11 supplementation reverses this effect, facilitating M2 polarization and alleviating neuronal injury (11).

IL11 can also exert indirect immunomodulation. For instance, in non-CNS contexts, mesenchymal stem cells secrete IL11 to suppress CD4^+^ T-cell activation, reduce IFN-γ production, and mitigate T cell-mediated tissue injury (63–65). This suppression of pro-inflammatory T-helper responses could subsequently influence macrophage activation states within the CNS. While direct evidence for such a pathway in ICH is currently lacking, it represents a plausible mechanism by which IL11 could broadly shape the neuroimmune landscape.

It is noteworthy that IL11 exhibits context-dependent, bidirectional regulatory effects. In certain disease models, IL11 upregulation is associated with enhanced pro-inflammatory responses—for example, in fibrotic lung disease, as well as cardiac and hepatic disorders, IL11 activates stromal cells, promotes immune cell recruitment, and sustains chronic inflammation (6, 66, 67). In contrast, within the CNS, moderate IL11 expression exerts anti-inflammatory and neuroprotective effects (11). Thus, IL11-mediated regulation of immune cell activation and phenotype is influenced by cell type, microenvironment, and disease stage. This bidirectional property provides a theoretical basis for targeting IL11 signaling, while also emphasizing the necessity of tailoring IL11 modulation to specific pathological contexts in order to optimize anti-inflammatory and neuroprotective outcomes in conditions such as ICH.

#### 1.4.2 Role of IL11 in blood–brain barrier protection

IL11 plays an important role in regulating post-ICH inflammatory responses and protecting the BBB. The evidence comes from both direct permeability assays (e.g., reduced Evans blue or IgG extravasation) and observations of enhanced tight junction protein expression in ICH models treated with IL-11 (16). The BBB is critical for maintaining CNS homeostasis, and its disruption allows plasma components and immune cells to infiltrate brain tissue, exacerbating injury. IL11 promotes BBB protection and repair through multiple mechanisms. First, it regulates cerebral microvascular endothelial cell function by enhancing the expression of tight junction proteins such as claudin-5 and occludin, thereby limiting paracellular permeability and preserving the structural integrity of the BBB (68, 69). Additionally, IL11 suppresses the excessive release of pro-inflammatory cytokines such as TNF-α and IL-1β, mitigating inflammation-induced BBB disruption.

Animal studies further support IL11’s protective role. In ICH models, exogenous IL11 administration significantly attenuated cerebral edema and reduced BBB structural damage. Treated animals exhibited higher expression of tight junction proteins, reduced BBB permeability, and decreased brain water content compared with controls—collectively indicating that IL11 ameliorates secondary injury after ICH. Moreover, IL11 may indirectly promote BBB repair by modulating glial activation and dampening secondary inflammation. Collectively, IL11 not only acts directly on BBB structure but also exerts protective effects through anti-inflammatory pathways, highlighting its potential as a therapeutic target for BBB preservation in ICH.

While IL-6, CNTF, and IL-11 all activate STAT3 and can support neuronal survival, their roles in BBB regulation differ. IL-6 is often associated with increased BBB permeability and neuroinflammation, whereas CNTF’s primary effects are more strongly linked to neuronal and astrocytic survival rather than direct vascular regulation. In contrast, the body of evidence in ICH and related models specifically points to a direct, protective role for IL-11 on BBB function, potentially representing a unique feature among gp130-signaling cytokines in this pathological context.

#### 1.4.3 IL11 and neuronal survival and repair

IL11 has recently gained attention for its role in neuronal survival and repair following CNS injury. Studies indicate that IL11 activates key signaling cascades, including STAT3 (70), to regulate downstream gene expression, thereby exerting anti-apoptotic and reparative effects. In ICH and related CNS injuries, neurons often undergo apoptosis or functional impairment due to oxidative stress and inflammation. By binding to its receptor and activating JAK/STAT3 signaling, IL11 upregulates anti-apoptotic genes such as Bcl-2 while suppressing pro-apoptotic factors such as Bax, thus reducing neuronal injury. Additionally, IL11 modulates glial function and the inflammatory microenvironment, creating conditions favorable for neuronal survival and regeneration (70, 71).

Regarding neural repair, IL11 not only suppresses aberrant cytokine release and attenuates local inflammation but also promotes axonal regeneration. Experimental evidence shows that IL11 treatment enhances neuronal viability and neurite outgrowth, supporting neural network reconstruction. Furthermore, IL11’s ability to reduce glial-derived neurotoxic factors and suppress secondary neuronal injury further facilitates recovery. By improving BBB repair and reducing cerebral edema, IL11 may also indirectly protect neurons. Figure 1 presents an integrative model of IL-11-mediated neuroprotection and immunomodulation following ICH.

As our understanding of IL11 biology deepens, its potential in neuroprotection and functional recovery becomes increasingly apparent. Future studies dissecting IL11-mediated molecular mechanisms of neuronal survival and repair may provide new therapeutic strategies for post-ICH neurological dysfunction. Targeted IL11-based interventions may ultimately advance neuronal regeneration and functional recovery.

### 1.5 Research progress on IL11 as a therapeutic target for inflammatory responses after ICH

#### 1.5.1 IL11-related drugs and therapeutic strategies

In recent years, pharmacological approaches targeting IL11 have demonstrated therapeutic potential in various animal models, particularly in inflammatory and fibrotic diseases. Recombinant human IL11 (rhIL11) was originally developed to treat thrombocytopenia; however, clinical application revealed severe side effects, including acute left ventricular dysfunction (72). In the context of ICH and related CNS inflammation, IL11 has garnered growing attention. Both *in vivo* and *in vitro* studies suggest that exogenous IL11 supplementation modulates astrocyte–microglia signaling, reduces pro-inflammatory responses, and mitigates neuronal injury. For example, in SAE models, IL11 administration significantly alleviated LPS-induced neuronal injury, suggesting a neuroprotective role (11). Furthermore, modified IL11 proteins (e.g., PEGylated rhIL11) exhibited improved tolerability in animal models. Although high doses may induce immunogenicity, these reactions tend to resolve during recovery (73).

Beyond supplementation, IL11 receptor antagonists and neutralizing antibodies are being actively investigated. Anti-IL11RA antibodies have shown efficacy in suppressing inflammation and fibrosis in models of renal injury, cardiac overload, and hepatic damage by inhibiting ERK and STAT3 pathways (8, 74, 75). Although no animal studies have directly evaluated IL11RA antagonists in ICH, findings from other CNS disease models support their potential applicability.

Combination therapy is another promising avenue. Certain antifibrotic drugs (e.g., nintedanib, pirfenidone) differentially modulate IL11 signaling: nintedanib induces ER stress, whereas pirfenidone does not (76). Small-molecule inhibitors targeting IL11 downstream effectors such as ERK and STAT3 also indirectly regulate IL11-mediated inflammation, with JAK/STAT inhibitors demonstrating efficacy in preventing IL11-induced cardiotoxicity (72). In CNS disease and cancer immunotherapy, modulation of IL11 pathways has been linked to improvements in the immune microenvironment and cytokine storm suppression, offering translational opportunities (77, 78).

Overall, IL11-based strategies are evolving from direct supplementation or blockade toward combinatorial approaches with conventional anti-inflammatory and antifibrotic agents, aiming at precise regulation of inflammation and tissue repair. These advances provide new therapeutic opportunities for ICH, although safety, efficacy, and mechanistic validation remain to be fully established in CNS models.

#### 1.5.2 Advantages and challenges of IL11-targeted therapy

IL11 plays dual roles in inflammation regulation and tissue repair, making it a compelling candidate for therapeutic targeting after ICH. Preclinical evidence indicates that IL11-targeted interventions can mitigate inflammation, reduce fibrosis, and promote regeneration. For instance, in renal injury models, IL11 therapy reduced inflammation and fibrosis while promoting epithelial proliferation and parenchymal regeneration, thereby improving organ function (8). Similarly, IL11 inhibition has demonstrated antifibrotic and organ-protective effects in models of cardiac and hepatic fibrosis (79). These findings suggest that targeting IL11 may improve ICH prognosis by alleviating inflammation and promoting repair.

Nevertheless, several challenges complicate clinical translation. First, IL11 exhibits strong spatiotemporal specificity, exerting divergent effects depending on disease stage and tissue context. In some organs, IL11 promotes inflammation and fibrosis (53). Thus, therapeutic targeting requires precise timing and dosing to avoid exacerbating pathology. Second, the broad distribution and pleiotropy of IL11 signaling introduce risks of adverse effects, including systemic inflammation, fibrosis exacerbation, or immune dysregulation (79). While IL11 overexpression induces multi-organ pathology in animals, long-term safety and immunogenicity of IL11 inhibitors remain unclear. Therefore, although IL11 targeting holds theoretical promise for improving ICH outcomes, further studies are essential to elucidate mechanisms, optimize spatiotemporal intervention strategies, and ensure safety.

#### 1.5.3 Clinical translation and future directions

At present, research on IL11 in post-ICH inflammation and clinical application remains in its infancy, with limited clinical data available. Although preclinical findings highlight IL11’s potential in modulating inflammation and promoting repair, significant gaps remain before clinical implementation. The post-ICH inflammatory response involves multiple cell types and pathways, with IL11 exhibiting context-dependent, bidirectional effects. These complexities heighten the challenges of ensuring both efficacy and safety in clinical applications. Moreover, the absence of large-scale clinical trials evaluating IL11-targeted therapies in ICH limits their translational potential.

Future directions include high-quality basic and translational studies to delineate IL11’s biological functions, molecular targets, and mechanistic roles in ICH-associated inflammation. Advances in precision medicine and personalized therapy further underscore the need to tailor IL11 interventions based on patient-specific molecular signatures, receptor expression patterns, and inflammatory profiles. Personalized modulation of IL11 signaling could maximize therapeutic benefit while minimizing adverse effects. Additionally, emerging technologies such as gene editing and RNA interference hold promise for fine-tuned modulation of IL11 signaling, offering new avenues for drug development.

A critical step in translating IL-11 research to the clinic is the validation of its relevance in human ICH. While large-scale clinical studies specifically focusing on IL-11 in ICH are still needed, preliminary translational insights can be gleaned from related conditions. For instance, elevated levels of IL-11 have been reported in the serum and CSF of patients with other neurological injuries or inflammatory conditions, where they sometimes correlate with disease severity or specific inflammatory endotypes (80, 81). Translating these findings to ICH, future prospective clinical studies are imperative to determine whether peripheral or central IL-11 levels can serve as a reliable biomarker for predicting hematoma expansion, functional outcome, or guiding targeted anti-inflammatory therapy.

Furthermore, to comprehensively understand the role of IL-11 across different stroke subtypes, its potential divergent functions should be considered, given that ICH differs from ischemic stroke, subarachnoid hemorrhage (SAH), and intraventricular hemorrhage (IVH) in both etiology and initial injury mechanisms. For instance, the predominantly intraparenchymal location of ICH, as opposed to the subarachnoid or intraventricular spaces involved in SAH and IVH, exposes IL-11 to distinct cellular environments (e.g., differing cellular constituents, cerebrospinal fluid flow dynamics, and immune cell populations). Experiments in a mouse model of middle cerebral artery occlusion (MCAO) have demonstrated that IL-11 treatment can improve neurological function and reduce the cerebral infarct volume (82). Although research on IL-11 in SAH and IVH remains particularly limited, investigating its expression patterns and functional roles in these related yet pathophysiologically distinct conditions may reveal important context-specific functions. Comparative studies across experimental stroke models could also help delineate whether the neuroprotective, inflammatory, and fibrotic roles of IL-11 are universally conserved or uniquely adapted to the specific pathophysiology of ICH, thereby refining potential therapeutic strategies.

In summary, despite current challenges, IL11 remains a promising target for therapeutic intervention in ICH. Continued research is warranted to clarify its mechanisms, optimize therapeutic strategies, and bridge the gap from bench to bedside.

## 2 Conclusion

In recent years, the role of post-ICH inflammation in neuronal injury and repair has emerged as a central focus in neuroscience and clinical medicine. Within this context, IL11, as a key member of the IL6 family, exhibits unique and complex biological functions. A growing body of preclinical evidence demonstrates that IL11 not only regulates immune cell activation but also contributes to BBB preservation and neuronal repair. Its multilayered, multitarget regulatory network positions IL11 as an essential factor in both the pathological progression and functional recovery after ICH.

Importantly, IL11’s bidirectional regulatory properties provide new perspectives for understanding the complexity of ICH-induced inflammation. On one hand, IL11 suppresses excessive inflammation and mitigates secondary injury, offering neuroprotective potential during the acute phase. On the other, IL11 may, under certain conditions, promote glial activation and pro-inflammatory cytokine release—underscoring the need for balanced therapeutic strategies. Current knowledge regarding IL11 signaling pathways, cell type–specific actions, and crosstalk with other cytokines remains incomplete. This field is still in its early stages, requiring systematic basic research, robust animal models, and well-designed prospective clinical studies.

From a translational standpoint, IL11 represents a novel and promising target for inflammation modulation. Ongoing challenges include defining molecular regulation, optimizing delivery systems, and evaluating safety. Maximizing IL11’s neuroprotective effects while avoiding adverse outcomes will be key for clinical application. Precision medicine and individualized treatment paradigms offer opportunities to refine IL11-based interventions, potentially enabling multidimensional modulation of ICH pathology in combination with other inflammatory targets.

In conclusion, IL11 holds significant promise in the context of ICH-related inflammation and neuronal repair. Advancing IL11 research will require integration of basic and translational studies, interdisciplinary collaboration, and the establishment of robust platforms for evaluation. Through continued exploration of IL11 mechanisms, optimization of therapeutic strategies, and incorporation of emerging technologies, IL11 may ultimately represent a breakthrough in improving prognosis for ICH patients and advancing precision therapy for CNS disorders.
